# Patient and practitioner views on cancer risk discussions in primary care: a qualitative study

**DOI:** 10.3399/BJGPO.2021.0108

**Published:** 2022-01-12

**Authors:** David N Blane, Sara MacDonald, Catherine A O'Donnell

**Affiliations:** 1 General Practice and Primary Care, Institute of Health and Wellbeing, University of Glasgow, Glasgow, UK

**Keywords:** neoplasms, prevention, qualitative research, general practice, socioeconomic status, disparities

## Abstract

**Background:**

It is estimated that nearly 600 000 cancer cases in the UK could have been avoided in the past 5 years if people had healthier lifestyles, with the principle modifiable risk factors being smoking, obesity, alcohol consumption, and inactivity. There is growing interest in the use of cancer risk information in general practice to encourage lifestyle modification.

**Aim:**

To explore the views and experiences of patients and practitioners in relation to cancer prevention and cancer risk discussions in general practice.

**Design & setting:**

Qualitative study among patients and practitioners in general practices in Glasgow, UK.

**Method:**

Semi-structured interviews were conducted with nine practitioners (five GPs and four practice nurses, recruited purposively from practices based on list size and deprivation status), and 13 patients (aged 30–60 years, with two or more specified comorbidities).

**Results:**

Currently, cancer risk discussions focus on smoking and cancer, with links between alcohol and/or obesity and cancer rarely made. There was support for the use of the personalised cancer risk tool as an additional resource in primary care. Practitioners felt practice nurses were best placed to use it. Use in planned appointments (for example, chronic disease reviews) was preferred over opportunistic use. Concerns were expressed, however, about generating anxiety, time constraints, and widening inequalities.

**Conclusion:**

Health behaviour change is complex and the provision of information alone is unlikely to have significant effects. Personalised risk tools may have a role, but important concerns about their use remain, particularly in areas of socioeconomic disadvantage.

## How this fits in

Cancer risk prediction tools have been developed for use in general practice. At present, cancer risk discussions mostly take place (if at all) in relation to smoking and cancer, with the links between obesity and/or alcohol and cancer rarely discussed. There was support from patients and practitioners for the use of a personalised cancer risk tool as an additional resource. Concerns were expressed, however, about generating anxiety, time constraints, and widening inequalities, as those most in need of support are least likely to access it.

## Introduction

In the UK, nearly 600 000 cancer cases could have been avoided in the past 5 years if people had healthier lifestyles, with the principle modifiable risk factors being smoking, obesity, alcohol consumption, and physical inactivity.^1^ In theory, primary care can help prevent cancer by supporting health behaviour change related to these risk factors,^2^ utilising its strengths of contact, coverage, coordination, and continuity.^3^ In addition, GPs and practice staff understand the areas in which people live and the wider structural influences on their lives and choices, which is particularly important in areas of socioeconomic deprivation.^4^

Primary care has, however, had mixed success in supporting behaviour change to date. Brief interventions by GPs can support smoking cessation,^5^ problem drinking,^6^ and engagement with weight management.^7^ In addition, recent interventions have attempted to tackle multiple health behaviours,^8^ recognising the common clustering of risk factors, particularly in deprived areas.^9^ However, previous research has identified that barriers to behaviour change operate at patient, practitioner, and system levels.^10,11^ Future intervention development needs to account for both individual and structural factors. This is important for cancer prevention, as socioeconomic differences in cancer incidence are well documented,^12^ in part owing to higher prevalence of unhealthy behaviours in more deprived areas,^13^ but also owing to wider structural inequalities.^14^

A systematic review of cancer risk assessment tools in primary care suggested that they could improve patient intention to change diet and physical activity, without causing an increase in cancer-specific anxiety.^15^ A subsequent systematic review of reviews of personalised risk feedback (not just cancer risk) found presenting risk information on its own did not produce sustained behaviour change.^16^ Following this, researchers in Cambridge, England, developed a personalised cancer risk calculator and found that there was support for providing personalised cancer risk information in general practice, but that more resources were required to implement this provision.^17^ This study did not, however, take socioeconomic status into account; cancer prevention is likely to be more challenging in deprived areas.

It remains unclear how best to incorporate cancer risk information into primary care consultations, in terms of timing, content, and professionals involved, and what the unintended consequences might be. Research to date has taken place in relatively affluent populations, so the present study looked to explore the views of patients and practitioners in the less affluent context of the west of Scotland, where health literacy and life expectancy are lower and the prevalence of multiple unhealthy behaviours is higher.^18^

The aim of the research was to explore the views and experiences of primary care practitioners and patients in relation to cancer prevention, and cancer risk information sharing in primary care, focused on the principle modifiable risk factors of smoking, obesity, alcohol, and physical inactivity.

## Method

### Approach

A qualitative study involving semi-structured interviews with patients and practitioners in Glasgow, Scotland.

### Participants

In the first stage of the research, a purposive sample of GPs and practice nurses were recruited from a range of practices, based on practice characteristics of list size (small, medium, large) and deprivation status (low, medium, high deprivation, based on the percentage of patients living in the 15% most socioeconomically deprived postcodes identified by the Scottish Index of Multiple Deprivation [SIMD]) (see Table 1). Practices were contacted by letter, explaining the nature of the study and inviting participation. If interested, they were emailed further information and consent forms.

**Table 1.** Sampling frame of patients (in *italics*) and practitioners (in bold)

**Practice list size**	**Practice deprivation status** (percentage of patients living in 15% most deprived SIMD postcodes)		
	Low (<15%)	Medium (15%–40%)	High (>40%)
Small (<4000 patients)	**PNF3**	**GPM3**	**PNF4**
Medium (4000–8000 patients)		**GPM1,** **PNF2**, *F3, F4*	**GPF2,** **PNF1**, *M2, M3, F2, M8*
Large (>8000 patients)	**GPF1**, *F5, M6, M7*		**GPM2**, *M1, F1, M4, M5*

SIMD = Scottish Index of Multiple Deprivation.

In the second stage, patients aged 30–60 years from participating practices were recruited, if they had two or more of the five criteria associated with unhealthy behaviours: currently smokes, obesity, diabetes, hypertension, and coronary heart disease. People with a history of cancer or deemed unsuitable to take part by their GP were excluded. Participants were offered a £40 gift voucher for taking part. There was no pre-existing relationship between participants and researcher. Recruitment was supported by staff from the NHS Research Scotland (NRS) Primary Care Research Network, who conducted searches of electronic GP records for eligible patients.

### Data collection

**Figure 1.**
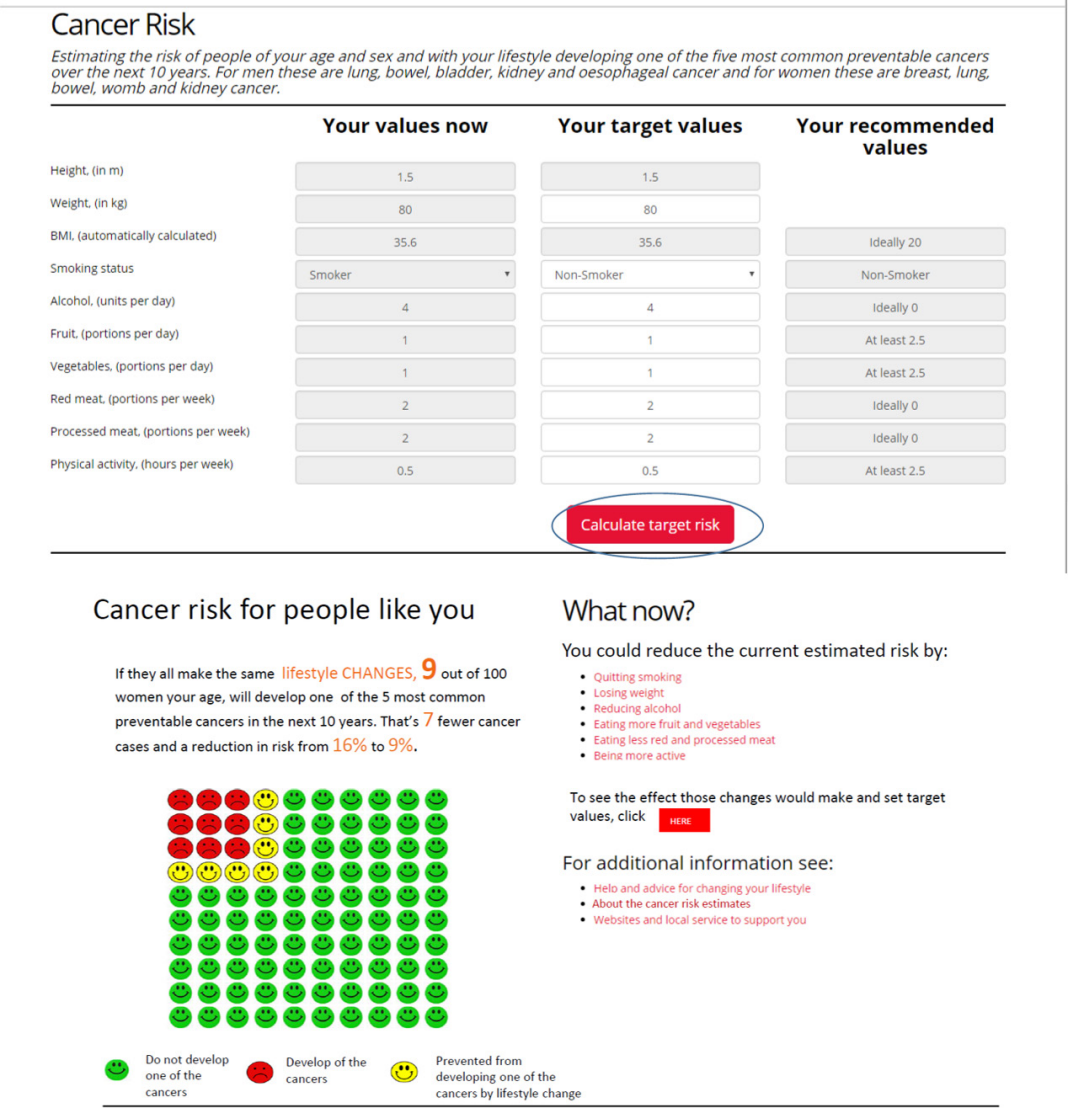
Screenshots of cancer risk tool^19^

An academic GP with expertise in qualitative research (DNB) conducted the interviews, which took place face to face at a time and place of the participant’s choosing, between December 2018 and August 2019. Interviews were audio-recorded with consent, and lasted between 30 and 55 minutes. They were semi-structured with the use of a topic guide, based on broad areas of interest (see Table 2). Screenshots from the cancer risk assessment tool developed by Dr Juliet Usher-Smith and colleagues^19^ were shared with permission to aid discussion (see Figure 1). Representative examples of potential patients (for example, a 50-year-old man with a body mass index [BMI] of 30 kg/m^2^) were discussed with participants, demonstrating typical reductions in cancer risk with adjustments to certain behaviours. Despite challenges with recruitment, data collection stopped when interviews were no longer generating new themes.^20^ The study was conducted and reported in accordance with the consolidated criteria for reporting qualitative research (COREQ).^21^

**Table 2.** Topic guide areas for patients and practitioners

**Patients**	**Practitioners**
Previous cancer risk discussions	Previous cancer risk discussions
Previous support for behaviour change	Role of primary care
Risk perception	Views on the risk tool (using screenshots and examples)
Views on the risk tool (using screenshots and examples)	Approach to supporting behaviour change
Role of primary care	Risk perception
Strategies to improve cancer prevention	Strategies to improve cancer prevention

### Analysis

Verbatim transcripts were checked, anonymised, and analysed inductively using a thematic approach.^22^ Analysis was done using NVivo (version 12) and led by DNB in collaboration with COD and SM. Interview transcripts were read and re-read, and a coding frame was developed after coding several transcripts. This was then systematically applied to each transcript. Themes and sub-themes were derived from this coding framework through an iterative process and then named to capture the ‘essence’ of what each theme was about.^22^ Differences between patients and practitioners, those from relatively more affluent and deprived areas, and between men and women, were considered within each theme.

## Results

Nine primary care practitioners (five GPs and four practice nurses) from eight different practices were interviewed (see Table 3), followed by 13 patients (see Table 4). There were four main themes: nuanced interpretations of cancer risk; difficulties discussing cancer risk in general practice; incorporating a cancer risk tool into consultations; and supporting behaviour change in deprived areas is hard.

**Table 3.** Practitioner characteristics

**Participant ID**	**Age range, years**	**Practice list size**	**Practice deprivation status** (percentage of patients living in 15% most deprived SIMD postcodes)
GPM1	50–60	Medium (4000–8000 patients)	Medium (15–40%)
GPM2	50–60	Large (>8000 patients)	High (>40%)
GPM3	50–60	Small (<4000 patients)	Medium (15–40%)
GPF1	50–60	Large (>8000 patients)	Low (<15%)
GPF2	50–60	Medium (4000–8000 patients)	High (>40%)
PNF1	50–60	Medium (4000–8000 patients)	High (>40%)
PNF2	50–60	Medium (4000–8000 patients)	Medium (15–40%)
PNF3	50–60	Small (<4000 patients)	Low (<15%)
PNF4	50–60	Small (<4000 patients)	High (>40%)

SIMD = Scottish Index of Multiple Deprivation.

**Table 4.** Patient characteristics

**Participant ID**	**Age range, years**	**Postcode deprivation (SIMD decile**)	**Health behaviours**	**Comorbidities**
M1	50–60	SIMD2	Used to smoke, obesity, physical inactivity, poor diet	Hypertension
M2	50–60	SIMD3	Used to smoke, obesity, poor diet	IHD, T2DM, sciatica
M3	40–49	SIMD1	Smokes, obesity	T2DM, OA, depression
M4	50–60	SIMD4	Obesity, physical inactivity	T2DM, hypertension, sciatica
M5	40–49	SIMD1	Smokes, occasional alcohol, obesity, poor diet	TIAs, hypertension, back pain
M6	50–60	SIMD8	Obesity, occasional alcohol	IHD, T2DM
M7	50–60	SIMD5	Smokes, obesity, physical inactivity, poor diet	T2DM, hypertension
M8	50–60	SIMD1	Smokes shisha pipe, obesity	Hypertension
F1	50–60	SIMD1	Used to smoke, obesity	IHD, T2DM, TIA, depression
F2	40–49	SIMD4	Smokes, excess alcohol, obesity, poor diet	T2DM, hypertension, depression
F3	50–60	SIMD7	Obesity	T2DM, hypertension
F4	50–60	SIMD9	Obesity, physical inactivity	T1DM, hypertension, hypothyroid
F5	50–60	SIMD6	Obesity	Hypertension, pre-diabetes

IHD = ischaemic heart disease. OA = osteoarthritis. SIMD = Scottish Index of Multiple Deprivation. T1DM = type one diabetes mellitus. T2DM = type two diabetes mellitus. TIA = transient ischaemic attack.

Illustrative data are provided to support the analysis, with data extracts identified by participant number, sex, and age range, as well as postcode deprivation status for patients (for example, M1, 50–60 years, SIMD2), and role and practice size for practitioners (for example, GPM1, 50–60 years, medium practice, medium deprivation).

### Nuanced interpretations of cancer risk

#### Understanding the causes of cancer

All participants were asked about their understanding of the causes of cancer and most recognised the influence of both genetic and environmental factors. Several patients quoted the statistic that one in two people will get cancer in their lifetime and noted that this had increased over time. Most put this down to a combination of lifestyle factors (smoking was still considered to be the leading cause) and changes in our food environment and air quality. Most practitioners had a similarly nuanced understanding of cancer causation, noting also that a decrease in cardiovascular mortality had resulted in more people living longer and, therefore, developing cancer.

In relation to the lifestyle factors in the cancer risk tool (Figure 1), several participants — both patients and practitioners — reported that they had only recently become aware of the links between obesity and cancer. Similarly, links between alcohol and certain cancers were not widely understood by patients. Practitioners were generally aware of these links, but did not discuss them routinely with patients, as described under the next theme.

#### Cancer risk perception and the role of chance

While recognising certain lifestyle factors as causes of cancer at a population level, both patients and practitioners highlighted the role of luck in whether a particular individual develops cancer, often relating stories of friends or family who had cancer while leading a healthy lifestyle (or smoked and drank heavily without developing cancer).

Typical cancer risk scores derived from the risk tool were discussed with participants, using illustrative examples. Practitioners were accustomed to using cardiovascular risk assessment tools, such as Q-Risk and ASSIGN, but it was noted that scores with cardiovascular risk tools were generally higher than those from the cancer risk tool. For instance, practitioners often discuss cardiovascular risk scores of 20–30% (that is, the chance of having a cardiovascular event in the next 10 years), but high scores from the cancer risk tool would typically be in the range of 5–10%. Participants were asked to reflect on these percentages:

Interviewer (INT): *'...* *say you heard that your risk over the next 10 years was 10% would that, would that resonate, do you think that would make you sit up and think? Or if they said it’s 5% would you think “well 5 out of 100, not much to worry about”?* *'*

M2: *'* *Well that would, I wouldn’t even say 10% would have been much to worry about, you’ve 90% chance you'll not get it but 10% chance, you know what I mean, it’s just a gamble isn't it, you know it really is a gamble, that’s it.* *'*

INT: '*And do you think that kind of timeframe, you know, thinking over the next 10 years do, do folk* *…* *'*

M2: *'* *I don’t really think that far ahead, I just take really each day by day, you know, we've got a saying “same shit, different day”.* *'* (M2, 50–60 years, SIMD3)

Others had a less fatalistic outlook and felt that any reduction in their cancer risk was worth working towards. Practitioners, too, had mixed views on typical percentage risk scores, with some recognising the potential for a motivational discussion based on even modest reductions in already low-risk percentages.

### Difficulties discussing cancer risk in general practice

#### Previous discussions focused on smoking

All participants were asked about previous cancer risk discussions. No patients could recall ever having discussed cancer risk in relation to health behaviours, although some did mention cancer screening conversations, suggesting how easily these can be conflated.

Most practitioners had only ever discussed cancer risk with patients in relation to smoking. This was partly related to not having the training or confidence to discuss links between cancer and risk factors such as obesity, but it was also clear that some GPs felt that discussing the spectre of cancer would not be a successful motivating factor to support behaviour change:

*'* *No it’s more in a kind of lifestyle positive way of a life affirming kind of change rather than "if you’re obese you’re going to get cancer and you die".* *'* (GPM3, 50–60 years, small practice, medium deprivation)

#### Risk advice must be delivered at an appropriate and sensitive time for the patient

For most participants, discussing cancer risk during a planned consultation (for example, a chronic disease review) felt more feasible than doing it opportunistically. Several practitioners emphasised the importance of context and timing:

*'* *It’s all got to be contextual, you’re not going to, you know, you're not going to start bringing up, you know, somebody’s had a bad life event and they're smoking more heavily or they're eating more, you’re not going to start, you know, their mother’s just died or they're, they’ve lost their job, you’re not going to start using that tool at that point. It’s a longitudinal, the relationship is longitudinal, the opportunities will occur.* *'* (GPM2, 50–60 years, large practice, high deprivation)

Practitioners were conscious that discussing health behaviours with patients can be sensitive, and that tone and language used was also important. A non-judgemental approach was advocated.

#### Practice nurses are best placed to discuss cancer risk

In keeping with the preference for planned discussions, most participants — patients and practitioners — felt that practice nurses were best placed to have cancer risk discussions. This was related to practice nurses having longer consultations and being more accustomed to using computer-based tools in their consultations, particularly with different templates for chronic disease reviews.

### Incorporating a cancer risk tool into consultations

#### Tool for motivating change or ‘tick-box’ exercise?

Most participants were enthusiastic about the tool, although there were notes of caution and patients were generally more positive about it than practitioners. Most patients felt the tool was easy to understand and that more knowledge was a good thing and could help motivate behaviour change.

Several recognised that tools such as this were not for everyone, and there is no 'one-size-fits-all' approach. Some patients reflected that using the tool in an interactive way during a consultation could help people prioritise behaviour changes that they wanted to make:

*'* *It’s giving you options rather than telling you “You need to do this, you need to do that”. So instead of cutting out smoking you could maybe eat more fruit and veg, eat less processed foods. So it would be more helpful because it gives you a chance to maybe do things one at a time rather than trying to do everything at once and it just blows up and you are like “oh, I can’t be bothered with it”.* *'* (M3, 40–49 years, SIMD1)

Other patients, however, were more sceptical, believing the tool to be impersonal, reducing individuals to a set of numbers.

GPs and practice nurses were mostly positive about the tool itself. One GP (GPF2), who was an advocate of ‘lifestyle medicine’, felt the tool could be helpful in motivating behaviour change. Another praised its content and layout. The main concern about the tool, over and above the potential unintended consequences covered later, was the potential for it to become a ‘tick-box’ exercise.

#### Collecting information in advance could help

Several GPs mentioned the possibility of gathering information for the tool in advance of the consultation, to save practitioner time and allow patients to consider priorities (if any) for health behaviour change. Practice nurses agreed that this approach could work for some patients, recognising the shift towards doing things online:

*'* *We are trying to get the patients to use the online website more and make appointments, ordering their prescriptions so that would be ideal.* *'* (PNF2, 50–60 years, medium practice, medium deprivation)

Patient and practitioners agreed this could also be done in the waiting room before appointments.

#### Beware unintended consequences

Participants were asked about potential unintended consequences of using a cancer risk tool in consultations. Practitioners raised more concerns than patients. Several GPs, for example, mentioned the possibility of generating anxiety, although this was less of a concern for the practice nurses in the study. Some patients also felt that there was the potential to increase worry, but this would depend on how the consultation was handled; if discussed sensitively, this would not be an issue.

A few GPs worried that using the tool would distract from the patient’s agenda and was not very person-centred; a concern shared by this patient:

*'* *I also think that sometimes what we do is we introduce tools for people who can’t have the conversation rather than teach people how to have the conversation. So should some of this money be spent in teaching GPs how to talk to patients or giving them enough space and time to talk to patients rather than having a* *"* *come and look at my computer* *"* *.* *'* (F3, 50–60 years, SIMD7)

Finally, a point made by several practitioners was that the tool could potentially widen health inequalities, as those most likely to benefit would be least likely to engage:

*'* *The inverse care law and the people that’s going to come and people that might change are the ones who are the, in the least need of change, the ones who are in the most need of change who, you know, but there’s nothing we can do about that in terms of, it’s a fact, we’ve just got to work round it.* *'* (GPM2, 50–60 years, large practice, high deprivation)

### Supporting behaviour change in deprived areas is hard

#### Barriers to behaviour change

Numerous barriers to behaviour change were discussed, with most being individual-level barriers shaped by socioeconomic circumstances. Several patients discussed the importance of individual willpower and motivation to make changes. For two patients, this motivation came from a sudden event such as a heart attack, which was a wake-up call to live a healthier lifestyle.

Some participants noted the importance of addressing underlying drivers of unhealthy behaviours, viewed by many as coping mechanisms. The addictive nature of many unhealthy behaviours was also raised, in relation to smoking, alcohol, and overeating:

*'* *Food is an addiction the same as heroin is an addiction but with food addiction, you need to take food to live. You can stop heroin because you don’t need heroin to live but you need food to live. So a food addiction to me is the worst addiction that people can have and it’s not so easy to stop and a lot of people just see it as a lifestyle choice. It’s not.* *'* (F1, 50–60 years, SIMD1)

Another factor was lack of time for physical activity, particularly for people working long hours, antisocial shift patterns, or doing several jobs to make ends meet. Language barriers (to accessing services) were also mentioned, along with cultural differences in health behaviours (for example, diet, smoking).

#### Need to address wider determinants of unhealthy behaviours

The most frequently cited determinant of unhealthy behaviours was poverty. It was noted that living in poverty affected a person’s ability to eat healthily and be physically active, and increased the likelihood of using smoking or alcohol as coping mechanisms. Some practitioners felt that people living in more deprived areas were less future-oriented — '*they live for the day* *'* (PNF1) — so were less likely to invest in their future health. In the quote below, this practice nurse, in an economically deprived area, pointed out that discussing fruit and vegetables would feel inappropriate in the context of more pressing demands such as mental health or addiction crises:

*'* *Sometimes it’s overwhelming. Really all I'm trying to do is to stop the alcohol or the* *… or the self-harming or the, it’s really, fruit and veg are way down the list. Sometimes you just think, you know, I'm not even mentioning that because it’s just insulting really, this person’s not got time for fruit or a vegetable.* *'* (PNF4, 50–60 years, small practice, high deprivation)

Commercial determinants of health were also discussed by patients and practitioners as having shaped environments to make people more sedentary and provide easier access to unhealthy energy-dense food and drink.

#### Helpful approaches to support behaviour change

Suggestions to improve support for health behaviour change ranged from the micro (individual) to the meso (community, institutional) to the macro (environmental, infrastructural) levels, although most focused on the role of primary care.

At the micro level, participants emphasised the importance of a person-centred approach in consultations:

*'* *We’ve got a bit into the* *… the house of care thing for diabetes, which is quite a nice model for actually trying to engage patients with what actually matters to them. And the whole thing that there is no point in telling someone to stop smoking if they actually want to die, you know, if you, you actually do need to try to engage with what people actually in practice can or want to do.* *'* (GPF1, 50–60 years, large practice, low deprivation)

At the meso level, patients described how various community resources had helped them to make health behaviour changes. Examples included smoking cessation support from pharmacies; support with changes to diet and exercise from groups, such as Slimming World or Weight Watchers; and support to reduce alcohol consumption from third-sector groups such as Alcoholics Anonymous. Many patients also said how helpful friends and family had been.

It was noted that many patients in deprived areas have other more pressing demands, so support with issues such as housing or benefits was considered a pre-requisite for engagement with health behaviour change. Community links practitioners made a big difference in this regard. One practice nurse said how helpful it was to have a welfare rights worker at the practice who could address financial concerns that were preventing engagement with health behaviour change.

Other factors that could support behaviour change at the practice level included more time in consultations; training (for GPs and nurses) in motivational interviewing and other approaches to supporting behaviour change; and better feedback from services that patients are referred to. Fruit and vegetable stalls in health centres were praised by one of the practice nurses.

Finally, at the macro level, action on the wider determinants of health behaviour was discussed. Most fundamentally, government intervention to tackle poverty was cited. Practitioners recognised that their actions to support health behaviour change were just one part of a multi-level approach, including advertising and changing social norms through positive role-modelling.

## Discussion

### Summary

This qualitative study of patient and practitioner views on cancer prevention and cancer risk information sharing in primary care found that cancer risk discussions, if they occurred at all, were about smoking and cancer, with links between alcohol and/or obesity and cancer rarely made. There was support for the use of the personalised cancer risk tool as an additional resource in primary care, although this was not universal. Concerns were expressed about generating anxiety, time constraints, and the potential for widening inequalities. Practitioners felt practice nurses were best placed to use the tool, as they had longer consultations and were used to incorporating tools in chronic disease reviews. Use in planned appointments was preferred over opportunistic use, as long as a person-centred approach was used.

Both patients and practitioners recognised the additional challenges of supporting health behaviour change in areas of socioeconomic deprivation, where unhealthy behaviours often co-exist alongside mental health problems, addictions, and other social issues. Practitioners described the advantages of having practice-attached staff, such as community links workers or welfare rights workers, who could address some of these more pressing concerns.

### Strengths and limitations

This is the first qualitative study that the authors are aware of that has explored the views of patients and practitioners about cancer prevention and the use of cancer risk tools in primary care consultations in areas of high deprivation.

A limitation of this study is that, owing to difficulties recruiting practitioners to take part, there were relatively few practices represented and the patients recruited were from only four of these practices. Participants were mostly from the oldest age group (50–60 years); all practitioners and all but three patients were in this category. It is possible that patients in younger age groups (for example, 30–39 years) may have different interpretations of their cancer risk and of risk discussions in general practice. Similarly, younger practitioners may have different views and experiences, which might affect generalisability of the findings.

This research was conducted before the COVID-19 pandemic was declared in March 2020. Since then, general practice has shifted to ‘remote by default’ with triage of appointments now the norm.^23^ It is unclear how this reconfiguration of general practice will impact opportunities for cancer risk discussions, or what the views of patients and practitioners would be about having risk discussions remotely.

### Comparison with existing literature

Findings from this study resonate with previous work in relation to cancer candidacy.^24^ Smoking was most strongly associated with cancer risk, as was increasing age, while the links between other health behaviours (such as alcohol consumption, physical inactivity, and poor diet) and cancer were less commonly made. In keeping with previous research, the uncertainty and unpredictability of cancer was also expressed by participants in this study, with examples of both the ‘unwarranted survivor’ and ‘anomalous death’ stereotypes.^25^

In contrast to previous research in this area, this study recruited participants predominantly from areas of concentrated socioeconomic deprivation. Of the nine practitioners involved, only two were from practices categorised as ‘low deprivation’. Of the 13 patients, four were from the most deprived decile (SIMD1), with only four from SIMD6 or above. The enthusiasm for using a cancer risk prediction tool, which was found in in previous studies,^17,26^ was not matched to the same extent in the present study, as participants highlighted the challenges for health behaviour change facing those living in poverty or in areas of socioeconomic deprivation.

The more present-oriented nature of health behaviours in deprived areas has been well documented,^27^ although underlying explanations for this are contested.^28^ Practitioners in economically deprived areas emphasised the importance of addressing more pressing needs, such as housing and benefits issues, before discussing cancer risk and behaviour change. The authors of the present study believe the concept of ‘prevention burden’ — recognising the ‘work’ involved in making and sustaining health behaviour change in different socio-cultural contexts — is worthy of further exploration in this regard.^29^

It has been said that *'* *relationships are the silver bullets of general practice and primary care* *'*,^30^ with evidence that GP empathy is closely related to patient enablement.^31^ Responders in this study would support this idea, recognising that opportunities for supporting behaviour change arise over time, and did not need to be forced into every consultation. Indeed, the possibility of jeopardising the valued person-centred approach in primary care — that the tool would be an *'* *intruder in the consultation* *'* ^32^ — was a significant concern.

### Implications for research and practice

Health behaviour change is complex, and the provision of cancer risk information alone is unlikely to have significant effects, particularly in deprived areas where there are numerous barriers to behaviour change.^16^ Further research to evaluate the implementation of personalised cancer risk tools within different primary care settings may be warranted, but important concerns about their use remain. Action on wider determinants of health and health behaviour is likely to be more effective for cancer prevention at a population level.^33^
